# Post-acute care referral in United States of America: a multiregional study of factors associated with referral destination in a cohort of patients with coronary artery bypass graft or valve replacement

**DOI:** 10.1186/s12911-019-0955-0

**Published:** 2019-11-14

**Authors:** Ineen Sultana, Madhav Erraguntla, Hye-Chung Kum, Dursun Delen, Mark Lawley

**Affiliations:** 1 Department of Industrial and System Engineering, Texas A&M University, College Station, TX USA; 2 Population Informatics Lab, Department of Health Policy and Management, School of Public Health, Texas A&M University, College Station, TX USA; 3 Department of Management Science and Information Systems, Spears School of Business, Oklahoma State University, Stillwater, USA

**Keywords:** Post-acute care, Patient discharge, Referral, Length of stay, Coronary artery bypass

## Abstract

**Background:**

The use of post-acute care (PAC) for cardiovascular conditions is highly variable across geographical regions. Although PAC benefits include lower readmission rates, better clinical outcomes, and lower mortality, referral patterns vary widely, raising concerns about substandard care and inflated costs. The objective of this study is to identify factors associated with PAC referral decisions at acute care discharge.

**Methods:**

This study is a retrospective Electronic Health Records (EHR) based review of a cohort of patients with coronary artery bypass graft (CABG) and valve replacement (VR). EHR records were extracted from the Cerner Health-Facts Data warehouse and covered 49 hospitals in the United States of America (U.S.) from January 2010 to December 2015. Multinomial logistic regression was used to identify associations of 29 variables comprising patient characteristics, hospital profiles, and patient conditions at discharge.

**Results:**

The cohort had 14,224 patients with mean age 63.5 years, with 10,234 (71.9%) male and 11,946 (84%) Caucasian, with 5827 (40.96%) being discharged to home without additional care (Home), 5226 (36.74%) to home health care (HHC), 1721 (12.10%) to skilled nursing facilities (SNF), 1168 (8.22%) to inpatient rehabilitation facilities (IRF), 164 (1.15%) to long term care hospitals (LTCH), and 118 (0.83%) to other locations. Census division, hospital size, teaching hospital status, gender, age, marital status, length of stay, and Charlson comorbidity index were identified as highly significant variables (*p*- values < 0.001) that influence the PAC referral decision. Overall model accuracy was 62.6%, and multiclass Area Under the Curve (AUC) values were for Home: 0.72; HHC: 0.72; SNF: 0.58; IRF: 0.53; LTCH: 0.52, and others: 0.46.

**Conclusions:**

Census location of the acute care hospital was highly associated with PAC referral practices, as was hospital capacity, with larger hospitals referring patients to PAC at a greater rate than smaller hospitals. Race and gender were also statistically significant, with Asians, Hispanics, and Native Americans being less likely to be referred to PAC compared to Caucasians, and female patients being more likely to be referred than males. Additional analysis indicated that PAC referral practices are also influenced by the mix of PAC services offered in each region.

## Background

Post-acute care (PAC) facilities provide treatment for acute-care patients following hospital discharge and are known to improve patient outcomes, readmission rates, mortality, and functional disability [1, 2]. Their usage has grown over 80% since 1996 [3], causing the U.S. Medicare’s annual PAC spending to double since 2001 [4]. Medicare spending on PAC for heart attack, congestive heart failure, and hip fracture grew 4.5–8.5% annually from 1994 to 2009, as compared to a growth of 1.5–2% per year for total spending in the U.S. [4]. Recent reports demonstrated PAC to be the largest contributor (40%) to Medicare spending variation among geographic regions [5]. For example, in 2013, Medicare spent one of every six dollars (about $60 billion) on PAC reimbursement [6].

Conditions frequently requiring PAC referral upon acute care discharge include respiratory failure, stroke, joint replacement, cardiac surgery, heart failure, and pneumonia. Services are provided to these patients through various settings, which include long-term acute care hospitals (LTCH, 428 facilities in the U.S.), inpatient rehabilitation facilities (IRF, 1165 in the U.S.), skilled nursing facilities (SNF, 16,000 in U.S.), and Certified Home Health Agencies (HHA, 33,000 in U.S.) [7]. Although these facilities play an essential role in improving acute-care patient outcomes, they are somewhat heterogeneous, poorly coordinated with acute-care hospitals, and exhibit high regional variations in usage and availability [8]. Overall, PAC is perhaps the least understood portion of the U.S. healthcare continuum, and limited research was completed on PACs’ effectiveness for the conditions and settings mentioned above.

In 2007, Heinemann [9] called for research to establish an evidence-based practice for PAC rehabilitation outcomes. Since then, many studies focused on the associations between PAC, hospital length of stay (LOS), and readmission, especially for stroke patients. Sacks et al. [2] observed positive associations between the increased use of PAC, shorter average hospital LOS, and lower risk-adjusted readmission rates. Burke et al. [10] worked on readmission from PAC facilities, identifying risk factors (e.g., impaired functional status, increased acuity) and timing (30 and 100 days) for readmission risk. Other researchers focused on variables associated with the Functional Independence Measure (FIM) score and PAC referral [11–15]; important determinants of discharge location included memory and comprehension, living status, and social support. The relationship between readmission and discharge location was also investigated [16, 17], which indicated that strong hospital-SNF linkages [16] and high nursing care quality [17] could mitigate readmission rates. Some studies on stroke patients also highlighted the influence of regional and facility-level variation in post-acute settings and hypothesized facility-level variation influenced rehabilitation outcome more than geographic location variation [18].

In contrast to stroke, little works are present that addressed PAC referral for cardiovascular disease (CVD) patients. CVD is the leading cause of death in the U.S. [19], and patients with CVD often require rehabilitation after cardiac surgery [20], especially those undergoing coronary artery bypass graft (CABG) or heart valve replacement (VR) accounting for over 500,000 operations annually [21, 22]. Since these patients exhibit increased risks of additional cardiac events, studies focusing on PAC rehabilitation of these patients are essential for restoring the quality of life and mitigating mortality risk [23, 24]. However, most of the existing studies on CABG patients explored variables associated with readmission and reported postoperative infection, heart failure, and cardio-pulmonary complications as the most common risk factors [25–27]. A Massachusetts study reported that readmission rates and patient mortality were held constant when significant reductions in acute-care LOS were accompanied by increased PAC usage [28]. Few others predicted hospital readmission following heart failure [29–31]; significant predictor variables included the type of valve surgery, hospital LOS, discharge location, age, and the degree of patient follow up.

So far, only a limited number of studies focused on CVD patients’ relation with PAC referral following acute-care discharge. Brown et al. [24] noted that 56% of the coronary artery patients of their study were referred for rehabilitation at discharge; patients exhibiting non-ST-segment elevation myocardial infarction, comorbidities, and greater age being more likely to be referred. Dolansky et al. [20] reported the prominent factors associated with PAC use for aged cardiac patients are: race, gender, and LOS. However, none of the studies on PAC referral of CVD patients incorporated detailed hospital information, i.e., location, capacity, and other specialties in their analysis so far. The integration of these information along with patient demographic and clinical data would be useful to achieve a more detailed understanding of the drivers influencing the variation in PAC referral practice.

Previous studies highlighted that PAC referral practices vary widely [32, 33], and it led many to believe that standardized referral protocols will be beneficial from both outcome and cost perspectives [2]. However, no uniform guidelines have been established yet to help providers predict the appropriate PAC destination for CABG and VR patients. To achieve this target, first, it is imperative to understand prevailing PAC referral practices across the dimensions of geographic region and patient acuity. Considering this target and the gaps as mentioned above in the literature, the objective of this study is to examine the geographic variations in PAC referral pattern and identify the associated risk factors related to hospital characteristics, patient demographics, and clinical information for the decision of discharge location for CABG and VR. To accomplish the objective, patient-level detailed cohort data was obtained through an electronic health record system, and the association of the risk factors influencing PAC as a discharge destination was quantified. Details of the analysis, results, and discussions are presented in the following sections.

## Methods

### Data source

Data was extracted from the Cerner Health Facts data warehouse, which was shared by the Oklahoma State University Center for Health System Innovation (OSU-CHSI). Cerner Health Facts data warehouse is a Health Insurance Portability and Accountability Act (HIPAA) compliant, geocoded data warehouse containing comprehensive clinical records related to pharmacy, laboratory, admission, and billing collected from participating clinical facilities starting from 2000 [34]. The Cerner’s HealthFacts data warehouse contains electronic medical records for over 63 Million unique patients obtained from more than 400 U.S. Cerner clients (hospital systems). This, de-identified fully HIPAA compliant relational database covers 16 years of longitudinal episodes, connected at the patient level using a unique patient identifier. The data contains information on patient specifics, hospital specifics, doctor specifics, diagnostic/clinical information, lab, pharmacy, and billing data.

### Study design

The study was a retrospective record review of 5 years’ EHR data spanned from January 2010 to December 2015 collected from the Cerner’s HealthFacts data warehouse. This data warehouse is donated to the Center for Health Systems Innovation at Oklahoma State University for medical and healthcare-related academic research. The data warehouse organized the electronic records in tables and relationships that readily allows for data extraction on ICD-9 procedure codes. The data is extracted by one of the co-authors on this paper (he is also the research director for the research center that provided the data). This study was reviewed by the Institutional Review Board of Texas A&M University and approved as an exempt study (IRB reference number IRB2016-0453 M).

### Study population

The study population consisted of patients who had CABG or valve replacement (*n* = 14,224) surgery. International Classification of Diseases, 9th Clinical Modification (ICD-9-CM) procedure codes (10 codes) [36.10–36.17, 36.19–36.2] were used to identify the CABG patients and (21 codes) [35.00–35.04, 35.10–35.14, 35.20–35.38, 35.97, 35.99] were used to identify patients with valve replacement. In case a patient had multiple hospitalizations for the same condition in the study window, only the first admission was included to avoid potential effects of aging or readmission.

**Fig. 1:**
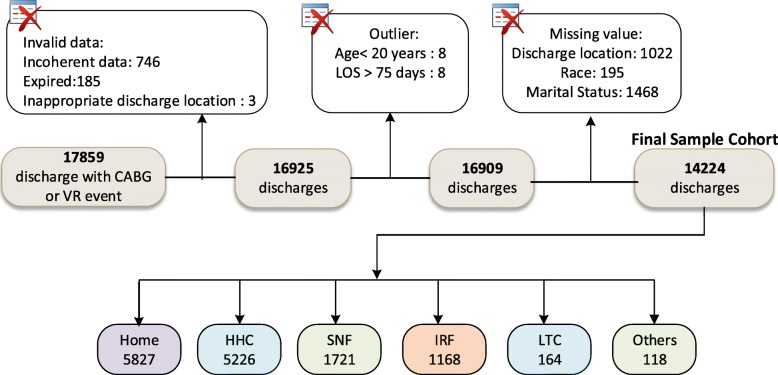
Flow diagram of the final sample cohort processing through data analysis

The study population was individuals with CABG or valve replacement and who were discharged alive after their index hospitalization. Patients who expired (*n* = 185), left against medical advice (LMA) or discharged for outpatient service were excluded from the study. The outpatient service was considered as inappropriate for this study because this study focuses on the referral to post-acute care facilities of the patient who got admitted and stayed in the acute care hospital for some days to get the required procedure done. Patients who had procedures performed before the admission date or after the discharge date (considered as incoherent data) were also excluded. These entries were considered incoherent data indicating data collection error because clearly it is not possible to have a procedure performed before admitting the hospital or after the discharge from the hospital. This study only included adult patients (> = 20 years) admitted through the emergency department or transferred from other clinical facilities or referred by a physician/HMO. This study excluded patients with length of stay > 75 days (*n* = 8) and age < 20 years (*n* = 8). Patients with missing predictor variables (*n* = 2685) were excluded. All these exclusions resulted in a final sample size of 14,224 patients from 49 acute care hospitals. Figure 1 summarizes the data cleaning and study cohort generation process.

### Definitions and variables

The dependent variable, discharge destination, was obtained from the initial encounter table. The categories of discharge destination were: (1) Discharged to home, (2) Discharged to home health care service (HHC) (3) Discharged to skilled nursing facility (SNF) (4) Discharged to long term care hospitals (LTCH) (5) Discharged to inpatient Rehabilitation facility (IRF) (6) Discharged to others. Discharged to ‘others’ included several miscellaneous discharge locations, and the number of patients discharged to these locations was very low. The miscellaneous locations were discharged to another short-term hospital, discharged within this institution to Medicare-approved swing bed, discharged to court/ law enforcement/jail, discharged to a designated cancer center or children’s hospital, discharged to a federal health care facility, discharged to a psychiatric hospital, and unknown. All of these miscellaneous locations are binned into one category ‘others’ to bring clarity in our analytical model.

In the analytical model, in total, 29 independent variables were considered. The independent variables were categorized into five categories like hospital location (census region), provider/hospital characteristics, patient demographics, related factors of PAC referral discharge, and comorbidity and diagnosis information. Table 1 provides a list of the 29 variables considered in this study.

**Table 1:** Variables considered for the discharge location analytical model

Category	Predictor Variables
Regional (Hospital Location)	Census division
Provider/Hospital	Bed Size Range
	Teaching Facility Affiliation
	Hospital Status
Patient Demographic	Race
	Gender
	Age
	Marital Status
Related factors of PAC referral discharge	Length of Stay
	Charlson Index
Comorbidity and Diagnosis information	Coronary Bypass of One Coronary Artery
	Coronary Bypass of Two Coronary Arteries
	Coronary Bypass of Three Coronary Arteries
	Coronary Bypass of Four or More Coronary Arteries
	Open Aortic Valve Replacement Tissue Graft
	Open Aortic Valve replacement
	Diabetes Mellitus without complications
	Tobacco Use disorder
	Atrial Fibrillation
	Unspecified Hypertension
	Coronary Atherosclerosis
	Intermediate Coronary Syndrome
	Hyperlipidemia
	Posthemorrhagic Anemia
	Acute Myocardial Infarction
	Congestive Heart Failure
	Anemia, Unspecified
	Pulmonary Collapse
	Acute Kidney Failure, Unspecified

This study considered census division of the hospital in the regional (hospital location) category and hospital bed size range, the teaching facility affiliation, and Hospital status (urban or rural) are categorized under provider/hospital characteristics. Demographic variables include age, marital status (married, divorced, single and widowed), race (Caucasian, African American, Asian, Hispanic, Native American and others), gender. Other predictor variables related to PAC referral discharge were the length of stay and the Charlson Index. The length of stay in the hospital was identified by the number of hospital days.

Based on the frequencies of the CABG and VR procedures in the study population, we identified four CABG procedures and two VR procedures, accounting for approximately 90.9% (12935) patients’ reasons for hospitalization. These include coronary bypass surgery for two arteries (*n* = 4496), coronary bypass surgery for three arteries (*n* = 3133), coronary bypass surgery for one artery (*n* = 2743), coronary bypass surgery for four or more arteries (*n* = 1108), open and other replacement of aortic valve with tissue graft (*n* = 874), and open and other replacement of aortic valve (*n* = 581). These six events were considered as independent binary variables to facilitate examination of the effect of these specific cardiac procedures in the discharge decision.

Comorbidity is defined as the coexistence of additional diseases or disorders in the same person with a specific index disease [35]. To assess the contribution of comorbid conditions in the discharge location (PAC referral), we examined if the patient had suffered from atrial fibrillation (ICD9–427.31), hypertension (ICD9–401.9), coronary atherosclerosis (ICD9–414.01), intermediate coronary syndrome (ICD9–411.1), hyperlipidemia (ICD9–272.4), acute posthemorrhagic anemia (ICD9–285.1), acute myocardial infarction (ICD9–410.71), tobacco use disorder (ICD9–305.1), diabetes mellitus without complication (ICD9–250), acute kidney failure (ICD9–584.9), pulmonary collapse (ICD9–518), congestive heart failure (ICD9–428) and unspecified anemia (ICD9–285.9). These 13 comorbid diagnoses were selected for assessment because they were the most frequent common comorbidities in the study population. However, the Charlson comorbidity index was used to capture the overall effect of comorbidities in each patient [36].

### Descriptive analysis and model development

**Fig. 2:**
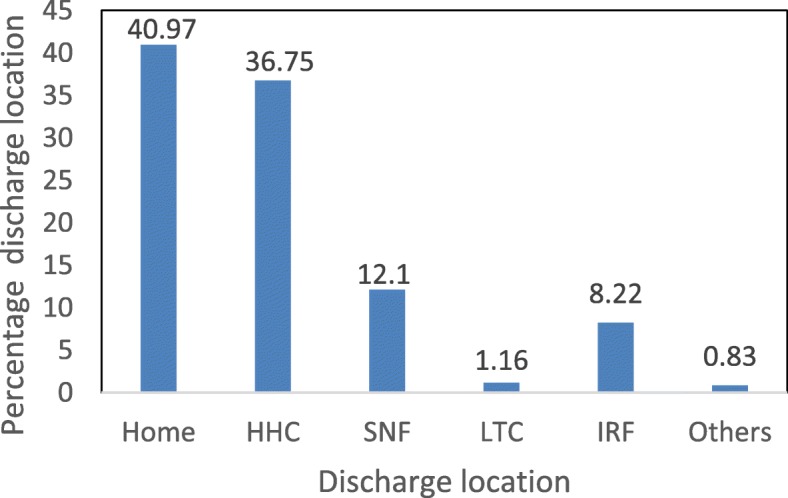
Distribution of discharge location

The primary focus of this study is the analysis of patient discharge location (PAC referral). Analyses included descriptive statistics for discharge location (Fig. 2) and exploratory analysis (univariate and bivariate analyses). Variables with large numbers of missing values and outliers were excluded. Chi-square tests were performed for categorical variables to test for differences in distribution of discharge locations among patients. Variables with *p*-value less than 0.1 [37] in the bivariate test were included as candidates in the multinomial logistic regression model. Percentages and medians with interquartile ranges are recorded for categorical and continuous variables in Table 3 in Appendix. The likelihood ratios for all variables are also reported in Table 4 in Appendix.

**Fig. 3:**
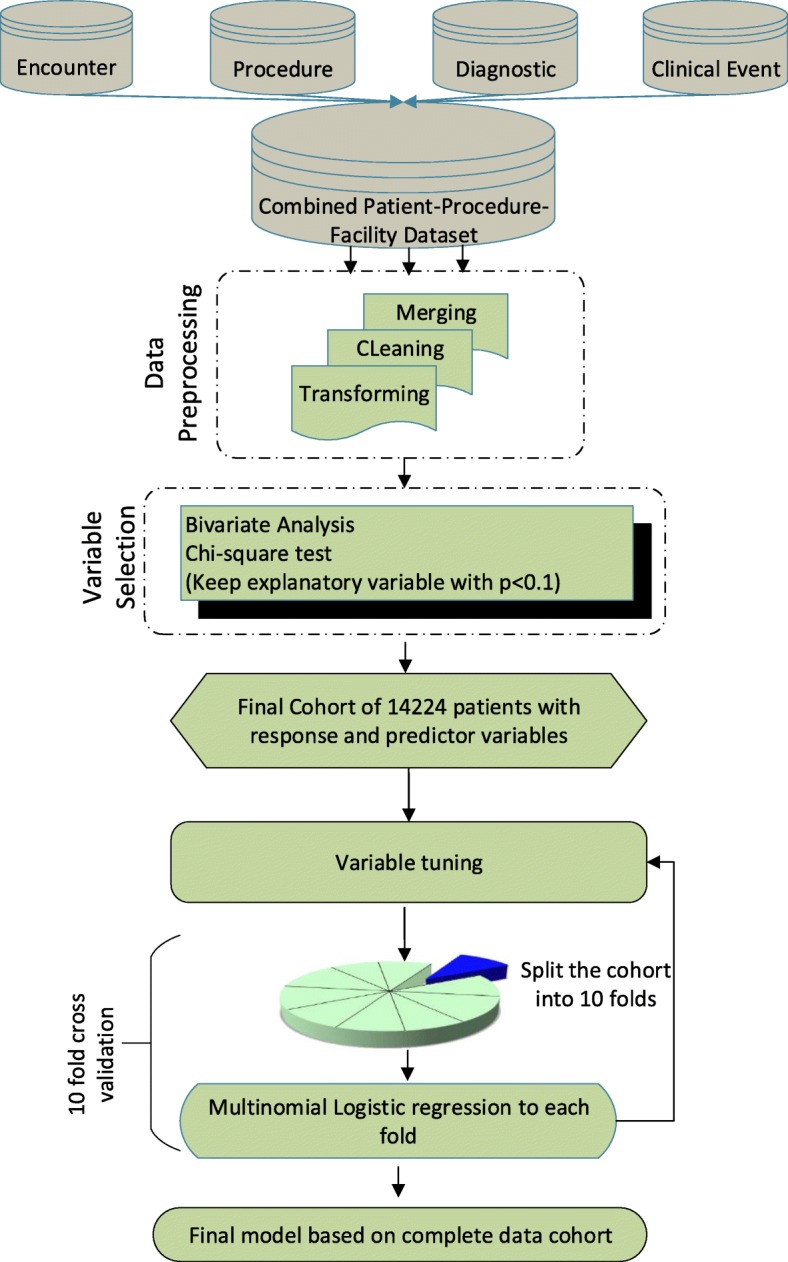
Flowchart of the methodology followed to develop the multinomial predictive model

Regression analysis has been widely used in healthcare and medical research in different predictive models specially in the field of disease prediction [38, 39], patient outcome prediction (i.e. readmission, mortality) [25, 27] and so on. Multinomial logistic regression is a popular method used for predicting a response variable with more than two categories (i.e. Home, LTCH, SNF, IRF, HHC). In this study, multinomial logistic regression was used to develop the analytical model, and ‘Home’ was used as the reference category. ‘Home’ was selected as the reference category because this category represented the highest percentage (40.96%) of the discharge destinations. To reduce bias in estimation of such analytical models, the clustering effect of patients within facilities within geographic regions is emphasized to consider performing multilevel mixed model [18, 40]. Therefore, we tested the multilevel analysis approach considering random effects from the census division and found that the difference between single and multilevel results is negligible. For example, the difference between single and multilevel model misclassification errors is 0.21% only. Also, the Akaike Information Criterion (AIC) difference between two models is 0.417%. It implies that there is none or minimal clustering effect of census divisions in our dataset. So, we only considered a single-level analysis approach in this work. The model’s accuracy was calculated based on multiclass receiver operating characteristic (ROC) value and overall misclassification error. A 10-fold cross-validation of the model was conducted to assess model overfitting. We contrasted the mean misclassification error of cross-validation with the misclassification error of the model developed with the entire cohort. A flowchart describing the methodology used to develop and validate the model is shown in Fig. 3.

The relative risk ratio (RR), the *p*-value, and the 95% likelihood confidence intervals of the predictor variables for each category are reported in Table 4 in Appendix section. The data analysis and all the statistical tests were carried out in R version 3.2.3, an open-source package from the R Foundation for Statistical Computing [41].

## Results

### Analysis and model interpretation

The final cohort of the study population had a mean age of 63.5 ± 11.81 years (mean ± sd) with 10,234 (71.9%) male and 11,946 (84%) Caucasian. Figure 2 describes the distribution of the discharge destination. The most dominant discharge location was Home (40.97%), followed by HHC (36.75%), SNF (12.10%), IRF (8.2%), and LTCH (1.16%). Table 3 in Appendix summarizes demographic characteristics, hospital information, and information related to cardiac events and comorbidities for each discharge location. Discharge location ‘others’ does not represent any specific PAC type. Therefore, in the rest of the result section, discharge location category ‘others’ is ignored while discussing the model insights.

#### Factors related to discharge destination selection

Table 4 in Appendix provides the significance of the factors associated with discharge destination in the multinomial logistic regression. A *p*-value of 0.05 was used as a threshold to distinguish significant variables. The relative risk ratio and 95% confidence interval (CI) limits are also provided in Table 4 in Appendix. The association of the factors related to discharge location is stated in the following paragraphs.

(i) Regional (Hospital Location)

**Fig. 4:**
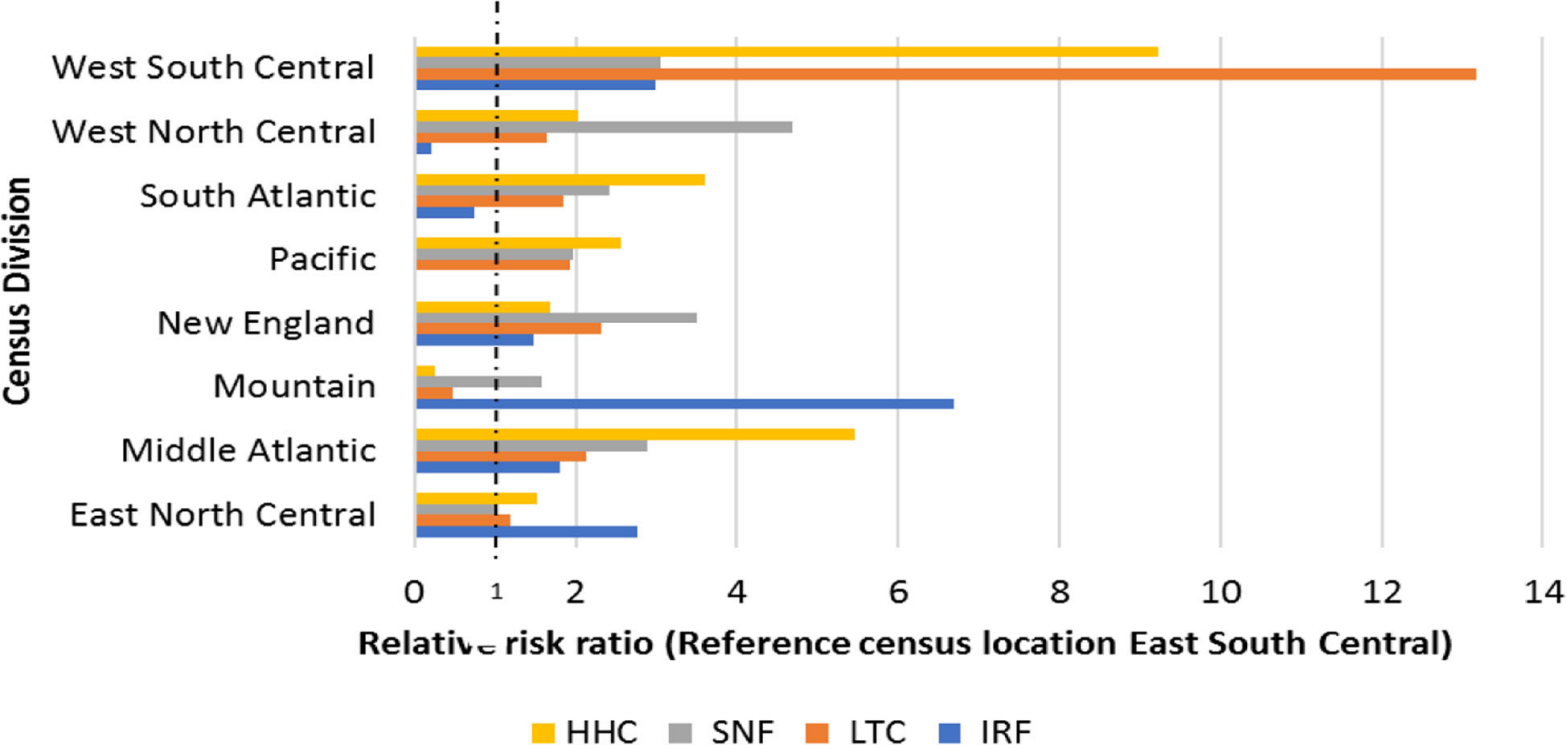
The relative risk ratio of 9 census divisions for 4 types of PAC

The location of the hospital, captured as census division, was found to be strongly associated with the selection of discharge location. For census division, ‘East South Central’ was chosen as the reference category. Patients in West South Central are around 13 times more likely to discharge to LTCH, 9 times more likely to HHC, and 3 times more likely to SNF or IRF compared to patients in East South Central. Patients from the Middle Atlantic are around 6 times more likely to discharge to HHC, 3 times to SNF, 2 times to IRF, and 2 times to LTCH compared to home than patients from East South Central. Figure 4 summarizes the risk ratios for the nine census divisions.

(ii) Provider/Hospital

**Fig. 5:**
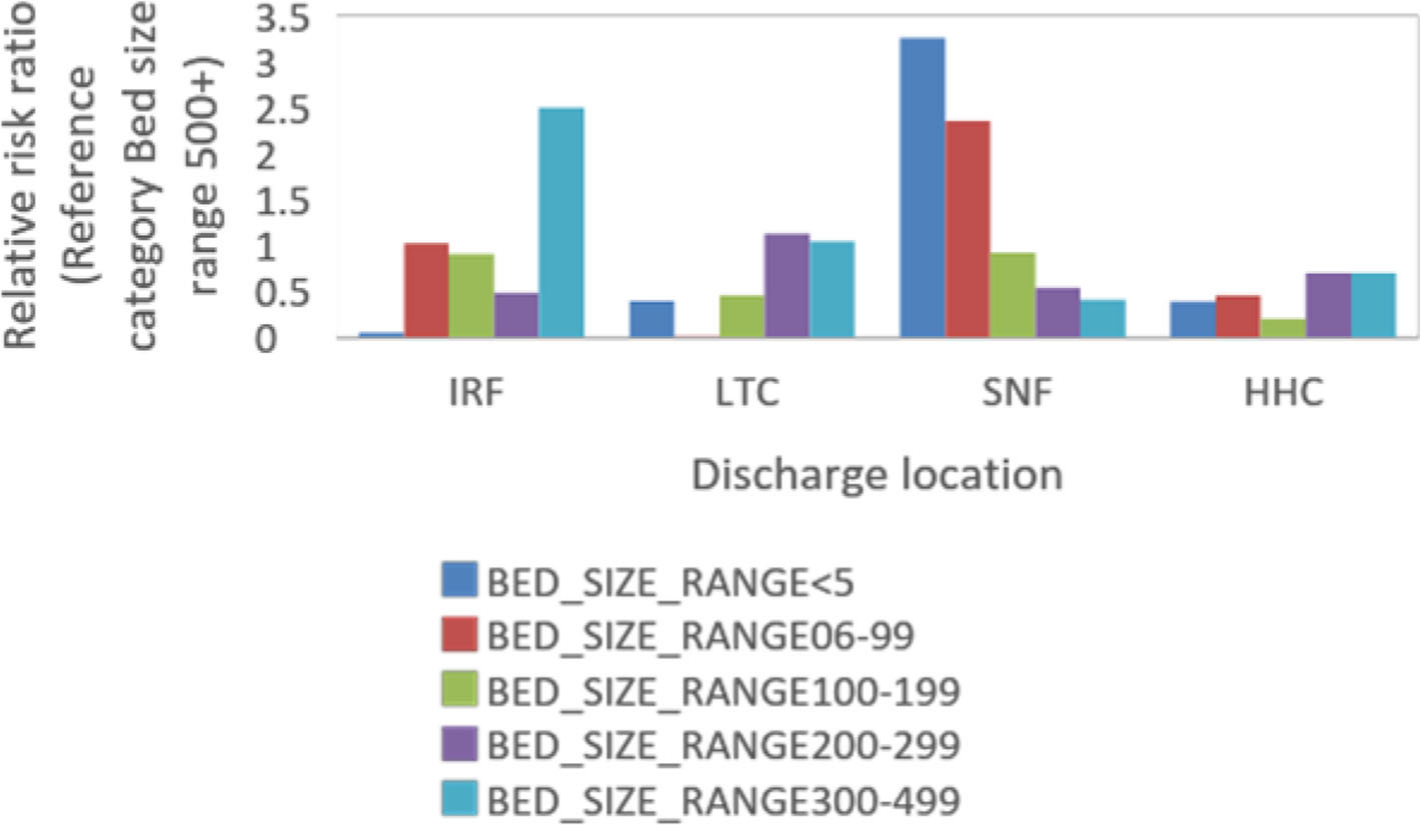
The relative risk ratio of different bed size range for 4 types of PAC

The bed size of the index hospital was also found to be a significant predictor of discharge destination. The bed size range of 500+ was chosen as the reference category in the model. Compared to the 500+ bed size hospitals, those with 300–499 bed size are 40 and 70% less likely to discharge patients to SNF and HHC, respectively, and around 3 times more likely to discharge to IRF compared to Home (Table 4 in Appendix). Patients from 200 to 299 bed size range hospitals are 50, 70, and 50% less likely to discharge to SNF, HHC, and IRF, respectively, compared to Home. Patients admitted to hospitals with bed size range 6–99 are less likely to be discharged to HHC and LTCH. Figure 5 summarizes the variation of the RR values for different bed size range.

Whether a facility is a teaching hospital or not was also a significant factor of discharge destination. Hospitals with teaching are less likely to discharge patients to PAC compared to home. No significant difference was found in referral to HHC, IRF, and LTCH between urban and rural hospitals.

(iii) Patient Demographic

Gender was found to be significant for discharge location. Females are more likely to be discharged to PAC than males. The likelihood of a female patient being discharged to SNF and LTCH is twice that of males. Further, Asians are around two times more likely to be referred to HHC compared to Caucasians, and single, divorced, and widowed patients are 2 to 3 times more likely to be discharged to SNF, IRF, and LTCH compared to married. Age is another significant predictor in the discharge destination referral, with the likelihood of PAC referral increasing with age.

(iv) Related factors of PAC referral discharge

Length of stay and Charlson comorbidity index were also significant predictor variables for the decision of discharge location. Patients with longer length of stay and higher comorbidity index were more likely to be released to a PAC facility compared to Home.

(v) Comorbidity and Diagnosis information

Patients with valve replacement exhibited higher discharge rates to PAC facilities compared to non-valve replacement. CABG and VR patients diagnosed with coronary atherosclerosis and tobacco use disorder are less likely to discharge to a PAC facility compared to Home. However, CABG or VR Patients diagnosed with acute kidney failure are 2 times more likely to discharge to LTCH. The associations of other individual comorbid diagnoses were not found to be significant.

### Predictive power of the model

**Fig. 6:**
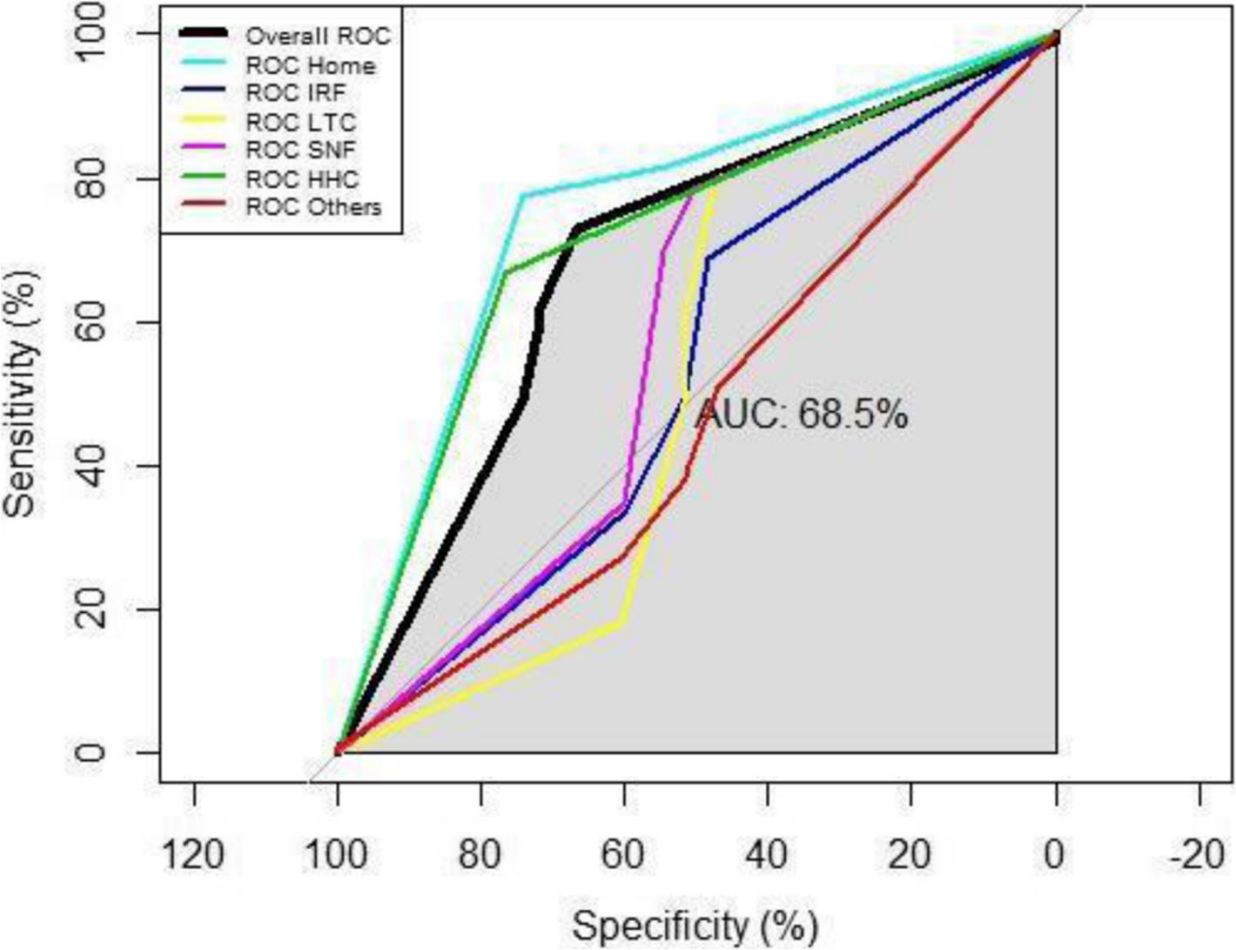
Receiver operating characteristics (ROC) curve for multiclass prediction model with multinomial logistic regression

The average 10-fold cross-validated predictive accuracy of the model is 62.6% considering overall misclassification error. The average cross-validation (CV) accuracy (62.5%) is consistent with the accuracy based on the complete cohort. The standard deviation of the accuracy based on CV is very low (0.015) indicating that model is very stable to data/sample variations. The misclassification error in each of the CV runs did not differ significantly from the findings in the complete cohort. Figure 6 shows the multiclass ROC curves for every discharge location category along with overall ROC for the model. pROC package from R was used to analyze and compare the multiclass ROC curves for 6 discharge locations [42]. The area under the overall ROC curve (AUC) is 0.685, and the AUC for Home, IRF, LTCH, SNF, HHC, and others are 0.72, 0.53, 0.52, 0.58, 0.72, and 0.46, respectively.

## Discussion

This study revealed that 54.5% of CABG patients and 73.3% VR patients were discharged with some PAC care. This finding seems reasonable because VR procedures are associated with more complexity than CABG. VR patients experience frequent complications after surgery that result in arrhythmias and unspecified heart failure [20]. For those receiving PAC, most were referred to HHC (relative proportion 63.1%), which is consistent with Dolansky et al. [20], who stated that surgery patients require less recovery care than non-surgical medical patients requiring lesser need for PAC. In our study population, IRF and LTCH were infrequently used as only 9.4% patients were sent to IRF and LTCH combined. This is also reasonable for the CABG or VR patients as they typically require little daily physical or occupational therapy (> 3 h) [20], which is a necessary admission criterion to discharge to IRF. Further, the average length of stay in our study population was 10 days, which does not meet the admission criteria of LTCH (more than 25 days for LTCH admission [43]).

Geographic variation of PAC use was significant, which is consistent with the existing literature [32, 33]. Picone et al. [44] hypothesized that the rate of PAC referral for cardiac patients aged 65 or more is positively correlated with the number of PAC facilities per 10,000 people, which our results partially support. Compared to other divisions (see Table 2), West South-Central exhibits higher relative capacity for both LTCH and HHC compared to the mean (LTCH: 2.27% vs 1.45%, HHC: 57.1% vs 37.83%) and higher relative referral to LTCH and HHC compared to the mean (LTCH: 1.80% vs 1.09%, HHC: 45.8% vs 31.59%). Similarly, West North Central exhibits both higher relative SNF capacity and relative SNF referral (69.73% vs 56.39, 20.4% vs 14.36%, respectively).

**Table 2:** PAC capacity and referral rate across census divisions

Census Divisions	Number of PAC Facilities (Percentage within Division)				Referral Percentage within Division				
	HHC	SNF	IRF	LTCH	HHC	SNF	IRF	LTCH	Home
East North Central	2486 (42.40)	3081 (52.55)	227 (3.87)	69 (1.18)	31.20	14.30	12.20	1.00	41.30
East South Central	443 (27.65)	1043 (65.11)	82 (5.12)	34 (2.12)	13.40	5.70	9.40	1.60	69.90
Middle Atlantic	619 (24.78)	1691 (67.69)	158 (6.33)	30 (1.20)	68.30	16.00	4.50	0.50	10.70
Mountain	765 (45.29)	794 (47.01)	99 (5.86)	31 (1.84)	2.50	17.30	1.90	0.30	78.00
New England	442 (30.84)	937 (65.39)	35 (2.44)	19 (1.33)	43.60	20.10	11.20	1.10	24.00
Pacific	1464 (45.26)	1630 (50.39)	117 (3.62)	24 (0.74)	27.30	21.20	0.00	1.20	50.30
South Atlantic	1842 (41.37)	2367 (53.17)	176 (3.95)	67 (1.50)	21.00	9.10	9.90	1.30	58.70
West North Central	770 (25.81)	2080 (69.73)	106 (3.55)	27 (0.91)	31.20	20.40	1.70	1.00	45.70
West South Central	3173 (57.1)	2026 (36.46)	232 (4.17)	126 (2.27)	45.80	5.10	9.70	1.80	37.60
Mean	37.83	56.39	4.32	1.45	31.59	14.36	6.72	1.09	46.24

Source: Capacities from Medicare, https://www.medicare.gov/; Referral rates from study data set taken from Cerner Health Facts Data Warehouse, https://business.okstate.edu/chsi/

However, this capacity effect does not always hold. For example, patients in the East North Central are more likely to be referred to IRF as compared to other divisions (12.2% vs 6.72%), even though the relative capacity is lower than average (3.87% vs 4.32%). Further, among divisions, Mountain exhibits high relative HHC capacity (45.29% vs 37.83%) with low relative HHC referral (2.5% vs 31.59%). For capacity and referral profiles within divisions, Middle Atlantic exhibits high SNF capacity (67.69%) with low SNF referral (16.00%) and low HHC capacity (24.78%) with high HHC referral (68.3%). Overall, these results strongly indicate that, while PAC capacities are sometimes positively associated with PAC referral, other significant underlying factors exist that may contravene the capacity effect. Although researchers conjecture causes such as practice styles, service quality, insurance coverage, and acute / PAC business relationships [32] for these underlying factors, geographic variation in PAC referral is not yet clearly understood.

Our findings suggest that hospital characteristics also affect PAC referrals significantly. Smaller hospitals are more likely to refer patients to SNF (Table 4 in Appendix Referent 500 beds: bed size < 5, 6–99: SNF RR ratios: 3.2, 2.3, respectively), while larger hospitals are more likely to refer to HHC (Table 4 in Appendix: bed size < 5, 6–99: HHC RR ratios; 0.4, 0.5, respectively). Teaching hospitals are less likely to refer to PAC across all PAC types (Table 4 in Appendix Referent Non-Teaching: Teaching Hospital: SNF RR: 0.2; HHC RR: 0.4; IRF RR: 0.4; LTCH RR: 0.1).

Length of stay and comorbidity are both correlated with PAC referral, which is consistent with past findings [32, 45, 46]. Hospital length of stay is important because early discharge can contribute to less control over the patient’s condition and more reliance on PAC use [32]. Our study indicates that total comorbidity (Charlson Index) is a better predictor than specific comorbid conditions. This means that overall health complexity has more influence on referral than individual comorbid conditions. As comorbidity increases, the patient is more likely to be referred to SNF, IRF, or LTCH than to HHC (Table 4 in Appendix Charlson: HHC RR 1.1; SNF RR 1.3; IRF RR 1.3; LTCH RR 1.3). This is consistent with studies on PAC referral for patients with stroke and hip replacement [45, 46]. However, tobacco users or patients with a smoking history are less likely to be referred to PAC, which contradicts the results reported by Brown et al. [24]. Our analysis also indicated that CABG and VR patients with coronary atherosclerosis are less likely to be discharged to the PAC facilities (SNF, IRF, LTCH).

Female patients are more likely to be referred to PAC than are males (Table 4 in Appendix Referent Male: RR > 1 for all PAC categories, SNF RR 2.0), which is consistent with Suaya et al. [47], and older patients are more likely to be referred to PAC than younger (Table 4 in Appendix Age: RR > 1 for all PAC categories, SNF RR 1.1). These results are consistent with the cardiac study of Dolansky et al. [20], but again contradict the findings of Brown et al. [24], who finds that younger cardiac patients are more likely to be referred to PAC (Age Referent < 50: 66–80 Odds Ratio (OR) 0.9; > 80 OR 0.7). We note that, although the average ages of our dataset and Brown’s are very similar, Brown et al. considers not only CABG and VR, but also myocardial infarction, percutaneous coronary intervention (PCI), stable angina, and heart transplant, which could account for these differences.

Race also influences PAC referral, with Caucasians being more likely to use SNF (Table 4 in Appendix SNF Referent Caucasian: Asian RR 0.4; Native American RR 0.2; Hispanic RR 0.9; African American RR 0.9); Asians and African Americans being more likely to use HHC (Table 4 in Appendix HHC: Asian RR 1.6; African American RR 1.2); and African Americans and Hispanics being more likely to use LTCH (Table 4 in Appendix LTCH: African American RR 1.6; Hispanic RR 1.2). Our results are generally consistent with the review of Cortes and Arthur [48], although they do not consider multiple categories of PAC. Explanations for these racial disparities in the referral practice include cultural practices, education, and language [48]. However, similar demographics-driven disparities are also observed in cardiac rehabilitation practice in Canada. Studies on cardiac rehabilitation referral on using Canadian dataset [49] also reported women, ethnocultural minorities and people with low income to be less referred for cardiac rehabilitation despite their higher needs.

This study is, however, not without limitations; major limitations include the following: First, we had limited information on patient socioeconomic status and potentially important variables such as discharge condition and discharge medication of the patients. This information can be used to validate the discharge locations referred to for patients in the dataset. Second, limited information was available regarding the PAC facilities; information on the PAC location, quality, and type of service, patient adherence, and length of stay at the PAC would have facilitated analysis of the patient metrics and outcome. Finally, since data on number of beds and health professionals working in the PACs were not available, the number of PAC facilities was used as a proxy variable for PAC capacity. A future extension to this study can consider true enrollment of patients into the PAC and analyze discrepancies between referral and enrollment patterns. Further studies should also include larger datasets, especially including more hospitals from each census region, and multilevel mixed modeling should be performed for the analysis to reduce the clustering effects of patients within facilities within geographic regions.

## Conclusion

In this study, factors linked to PAC referral following acute care discharge was investigated using an EHR-extracted CABG and VR patient dataset. Our findings concluded that the regional location of the hospitals and hospital capacity (bed size) influenced the patient discharge practice. Disparities in PAC availability vis-a-vis referral across different U.S. census regions were also observed. The racial and gender-based disparity was also statistically significant, with Asians, Hispanics, and Native Americans being less likely to be referred to PAC compared to Caucasians, and female patients being more likely to be referred than males. Though patients diagnosed with relevant comorbid conditions were, in most cases, likely to be discharged to PAC facilities after the CABG or VR procedure, tobacco disorder and coronary atherosclerosis patients were less likely to be referred to PAC. These findings can help the clinicians to streamline the discharge planning process early in the patient’s acute care stay, and thereby, facilitate discharge processes, care coordination, and transition of care, following surgery. In future, inclusion of supportive information from the PAC facilities could allow to account for the PAC effectiveness and result in more robust and insightful findings.

## Data Availability

The datasets analyzed in this study were based on sensitive EHR data and is not publicly available to protect patient privacy. Researchers can contact corresponding author for details of obtaining the data use agreement to have access to the data.

## Appendix

**Table 3:** Summary statistic of the variables included in the multinomial logistic regression model across the discharge destinations

Variables	Discharged to						*p*-value
	Home	HHC	SNF	IRF	LTCH	Others	
Regional (Hospital location)							
Census Division:							
East North Central	704 (4.95)	548 (3.85)	252 (1.77)	215 (1.51)	17 (0.12)	21 (0.15)	< 0.001
East South Central	2007 (14.11)	390 (2.74)	166 (1.17)	274 (1.93)	47 (0.33)	19 (0.13)	
Middle Atlantic	329 (2.31)	2125 (14.94)	497 (3.49)	139 (0.98)	15 (0.11)	8 (0.06)	
Mountain	283 (1.99)	9 (0.06)	63 (0.44)	7 (0.05)	1 (0.01)	1 (0.01)	
New England	324 (2.28)	606 (4.26)	280 (1.97)	156 (1.10)	15 (0.11)	10 (0.07)	
Pacific	118 (0.83)	67 (0.47)	52 (0.37)	0 (0.00)	3 (0.02)	5 (0.04)	
South Atlantic	1039 (7.3)	382 (2.69)	165 (1.16)	180 (1.27)	24 (0.17)	30 (0.21)	
West North Central	324 (2.28)	226 (1.59)	148 (1.04)	12 (0.08)	7 (0.05)	7 (0.05)	
West South Central	699 (4.91)	873 (6.14)	98 (0.69)	185 (1.30)	35 (0.25)	17 (0.12)	
Hospital/Provider							
Bed Size Range:							
< 5	2 (0.01)	1 (0.01)	2 (0.01)	0 (0.00)	0 (0.00)	1 (0.01)	< 0.001
06–99	26 (0.18)	24 (0.17)	9 (0.06)	6 (0.04)	0 (0.00)	1 (0.01)	
100–199	758 (5.33)	309 (2.17)	243 (1.71)	124 (0.87)	8 (0.06)	12 (0.08)	
200–299	1204 (8.46)	502 (3.53)	232 (1.63)	125 (0.88)	39 (0.27)	26 (0.18)	
300–499	1221 (8.58)	976 (6.86)	362 (2.54)	372 (2.62)	28 (0.20)	32 (0.22)	
500+	2616 (18.39)	3414 (24)	873 (6.14)	541 (3.80)	89 (0.63)	46 (0.32)	
Hospital Status:							
Urban	5630 (39.58)	4602 (32.35)	1501 (10.55)	1064 (7.48)	161 (1.13)	110 (0.77)	< 0.001
Rural	197 (1.38)	624 (4.39)	220 (1.55)	104 (0.73)	3 (0.02)	8 (0.06)	
Teaching Facility Affiliation	4863 (39.5)	4069 (33.03)	1124 (9.1)	441 (3.6)	108 (0.9)	124 (1.0)	< 0.001
Patient demographic							
Gender:							
Male	4473 (31.25)	3874 (27.24)	980 (6.89)	728 (5.12)	95 (0.67)	84 (0.59)	< 0.001
Female	1354 (9.52)	1352 (9.51)	741 (5.21)	440 (3.09)	69 (0.49)	34 (0.24)	
Marital Status:							
Married	3832 (26.94)	3527 (24.80)	768 (5.40)	579 (4.07)	74 (0.52)	51 (0.36)	< 0.001
Divorced	682 (4.79)	483 (3.40)	227 (1.60)	156 (1.10)	27 (0.19)	16 (0.11)	
Single	881 (6.19)	691 (4.86)	293 (2.06)	168 (1.18)	25 (0.18)	36 (0.25)	
Widowed	432 (3.04)	525 (3.69)	433 (3.04)	265 (1.86)	38 (0.27)	15 (0.11)	
Race:							
Caucasian	4705 (33.08)	4524 (31.81)	1532 (10.77)	977 (6.87)	118 (0.83)	90 (0.63)	< 0.001
African American	868 (6.10)	455 (3.20)	137 (0.96)	155 (1.09)	37 (0.26)	23 (0.16)	
Asian	64 (0.45)	73 (0.51)	9 (0.06)	10 (0.07)	2 (0.01)	0 (0.00)	
Hispanic	38 (0.27)	24 (0.17)	6 (0.04)	8 (0.06)	2 (0.01)	1 (0.01)	
Native American	41 (0.29)	27 (0.19)	6 (0.04)	9 (0.06)	2 (0.01)	2 (0.01)	
Others	111 (0.78)	123 (0.86)	31 (0.22)	9 (0.06)	3 (0.02)	2 (0.01)	
Age in years	59.4 + 11	63.6 + 11.2	72.3 + 10.00	70.3 + 10.5	67.9 + 10.5	63 + 12.2	< 0.001
Related factors of PAC referral discharge							
Length of stay, days	7 [5,10]	7 [5,11]	11 [8,16]	12 [8,18]	25 [20,37]	11 [7,17]	< 0.001
Charlson index	2 [1,3]	1 [1,3]	2 [1,4]	3 [1,4]	3 [2,5]	2 [1,4]	< 0.001
Comorbidity and diagnosis information							
Coronary Bypass of Two Coronary Arteries	1757 (14.3)	1416 (11.5)	453 (3.7)	188 (1.5)	47 (0.4)	43 (0.3)	0.13
Coronary Bypass of Four or More Coronary Arteries	478 (3.9)	352 (2.9)	85 (0.7)	60 (0.5)	2 (0.02)	9 (0.1)	0.004
Coronary Bypass of Three Coronary Arteries	1423 (11.6)	915 (7.4)	256 (2.1)	126 (1.0)	23 (0.2)	31 (0.25)	< 0.001
Coronary Bypass of one Coronary Artery	1012 (8.2)	916 (7.4)	247 (2.0)	103 (0.8)	27 (0.2)	24 (0.2)	0.026
Open Aortic Valve Replacement Tissue Graft	161 (1.3)	269 (2.2)	153 (1.2)	45 (0.4)	11 (0.1)	18 (0.15)	< 0.001
Open Aortic Valve replacement	188 (1.5)	172 (1.4)	69 (0.6)	44 (0.4)	6 (0.05)	9 (0.1)	< 0.001
Diabetes Mellitus without complications	1800 (14.6)	1236 (10.0)	397 (3.2)	176 (1.4)	34 (0.3)	53 (0.4)	0.0002
Tobacco Use disorder	1588 (12.9)	848 (6.9)	163 (1.3)	80 (0.6)	22 (0.2)	31 (0.3)	< 0.001
Atrial Fibrillation	1415 (11.5)	1305 (10.6)	567 (4.6)	248 (2.0)	57 (0.5)	60 (0.5)	< 0.001
Unspecified Hypertension	3149 (25.6)	2601 (21.1)	656 (5.3)	257 (2.1)	42 (0.3)	77 (0.6)	< 0.001
Coronary Atherosclerosis	5418 (44.0)	4226 (34.3)	1199 (9.7)	538 (4.4)	104 (0.8)	132 (1.1)	< 0.001
Intermediate Coronary Syndrome	1431 (11.6)	867 (7.0)	220 (1.8)	100 (0.8)	11 (0.1)	28 (0.2)	< 0.001
Hyperlipidemia	3851 (31.3)	2734 (22.2)	752 (6.1)	349 (2.8)	51 (0.4)	87 (0.7)	< 0.001
Posthemorrhagic Anemia	1918 (15.6)	1415 (11.5)	531 (4.3)	249 (2.0)	60 (0.5)	56 (0.5)	< 0.001
Acute Myocardial Infarction	1233 (10.0)	885 (7.2)	351 (2.8)	161 (1.3)	40 (0.3)	35 (0.3)	< 0.001
Congestive Heart Failure	938 (6.6)	935 (6.6)	565 (3.9)	389 (2.7)	79 (0.6)	34 (0.3)	< 0.001
Anemia, Unspecified	807 (5.7)	707 (4.9)	263 (1.8)	211 (1.5)	29 (0.2)	20 (0.1)	< 0.001
Pulmonary Collapse	900 (6.3)	1330 (9.3)	368 (2.6)	239 (1.7)	46 (0.3)	24 (0.2)	< 0.001
Acute Kidney Failure, Unspecified	520 (3.7)	435 (3.1)	318 (2.2)	256 (1.8)	73 (0.5)	24 (0.2)	< 0.001

(All values listed as x(y) denote x = number of count, y = % for a particular discharge location; values listed as x + y denote x = mean and y = standard deviation; values listed as x [y,z] denote x = median, y = 1st quartile and z = 3rd quartile; *p*-values are generated from bivariate chi-square test)

**Table 4:** Risk ratios, *p*-values and 95% CI of the predictor variables in the model

Variables	Risk Ratio ^*p*-value^ (95% CI)							
	HHC		SNF		IRF		LTCH	
Regional (Hospital location)								
Census Division: East South Central (Reference)								
East North Central	1.5^***^	(1.3, 1.7)	1.00	(0.8,1.2)	2.8^***^	(2.0, 3.7)	1.2	(0.6, 2.1)
Middle Atlantic	5.4^***^	(4.7, 6.2)	2.9^***^	(2.3,3.4)	1.8^***^	(1.4, 2.2)	2.1^**^	(1.2, 3.7)
Mountain	0.3^***^	(0.1, 0.4)	1.6^**^	(1.1,2.1)	6.7^***^	(4.3,10.4)	0.5	(0.0, 2.5)
New England	1.7^***^	(1.4, 1.9)	3.5^***^	(2.8,4.2)	1.5^***^	(1.1, 1.8)	2.3^**^	(1.3, 4.1)
Pacific	2.5^***^	(1.7, 3.7)	1.9^***^	(1.3,2.7)	0.0^***^	(0.0, 0.0)	1.9	(0.5, 6.9)
South Atlantic	3.6^***^	(2.9, 4.5)	2.4^***^	(1.7,3.3)	0.8	(0.5, 1.0)	1.9	(0.8, 3.9)
West North Central	2.0^***^	(1.7, 2.4)	4.7^***^	(3.5,6.1)	0.2^***^	(0.1, 0.3)	1.6	(0.6, 3.9)
West South Central	9.2^***^	(7.6,11.1)	3.0^***^	(2.1,4.3)	3.0^***^	(2.2, 3.9)	13.2^***^	(6.7, 25.6)
Hospital/Provider								
Bed Size Range: 500 + (Reference)								
< 5	0.4^**^	(0.0,4.5)	3.3^**^	(0.2,38.1)	0.1^***^	(0.0,0.1)	0.4^***^	(0.3, 0.4)
06–99	0.5^*^	(0.2, 0.8)	2.4^**^	(0.8,6.24)	1.0^**^	(0.3, 2.8)	0.0^***^	(0.0, 0.0)
100–199	0.2^***^	(0.1, 0.2)	0.9^**^	(0.6,1.2)	0.9^**^	(0.6, 1.2)	0.5^**^	(0.1, 1.2)
200–299	0.7^***^	(0.5, 0.8)	0.5^***^	(0.4,0.6)	0.5^***^	(0.3, 0.6)	1.1^**^	(0.6, 2.1)
300–499	0.7^***^	(0.5, 0.8)	0.4^***^	(0.3, 0.5)	2.5^***^	(1.9, 3.2)	1.0^**^	(0.5, 2.1)
Hospital Status: Urban (Reference)								
Rural	1.1	(0.8, 1.4)	1.4^*^	(1.0,2.0)	0.9	(0.6, 1.4)	0.2	(0.1, 1.1)
Teaching Facility Affiliation	0.4^***^	(0.3, 0.4)	0.2^***^	(0.1,0.3)	0.4^***^	(0.3, 0.6)	0.1^***^	(0.1,0.3)
Patient demographic								
Gender: Male (Reference)								
Female	1.3^***^	(1.1, 1.4)	2.0^***^	(1.7, 2.3)		1.6^**^	(1.3, 1.9 1.9^**^	(1.2, 2.9)
Marital Status: Married (Reference)								
Divorced	1.0	(0.8, 1.2)	3.1^***^	(2.4,3.9)	2.3^***^	(1.8, 3.0)	3.2^***^	(1.8, 5.7)
Single	1.0	(0.9, 1.2)	3.4^***^	(2.7,4.2)	2.1^***^	(1.6, 2.7)	2.2^**^	(1.2, 4.1)
Widowed	1.1	(0.9, 1.3)	2.0^***^	(1.6,2.5)	1.9^***^	(1.5, 2.5)	2.6^***^	(1.5, 4.6)
Race: Caucasian (Reference)								
African American	1.2^*^	(1.0, 1.4)	0.9	(0.7,1.2)	1.00	(0.7, 1.2)	1.61	(0.9, 2.8)
Asian	1.6^*^	(1.0, 2.6)	0.4	(0.2,1.1)	0.67	(0.2, 1.5)	0.43	(0.0, 4.4)
Hispanic	0.6	(0.3, 1.2)	0.9	(0.3,2.8)	0.71	(0.2, 1.9)	1.24	(0.1,11.5)
Native American	0.5	(0.3, 1.1)	0.5	(0.2,1.4)	1.1	(0.4, 2.9)	0.0^***^	(0.0, 0.0)
Other	1.1	(0.8, 1.7)	0.8	(0.4,1.4)	0.64	(0.2, 1.4)	0.98	(0.1, 5.2)
Age in years	1.0^***^	(1.0, 1.0)	1.1^***^	(1.1,1.1)	1.1^***^	(1.0, 1.1)	1.1^***^	(1.0, 1.1)
Related factors of PAC referral discharge								
Length of stay, days	1.0^***^	(1.0, 1.0)	1.1^***^	(1.1, 1.1)	1.1^***^	(1.0, 1.1)	1.2^***^	(1.1, 1.2)
Charlson index	1.1^***^	(1.0, 1.1)	1.3^***^	(1.2,1.3)	1.3^***^	(1.2, 1.4)	1.3^***^	(1.1, 1.4)
Comorbidity and diagnosis Information								
Coronary Bypass of Two Coronary Arteries	1.0^***^	(0.8, 1.1)	1.0^***^	(0.8,1.3)	0.9^***^	(0.7,1.2)	1.6^***^	(0.9,3.0)
Coronary Bypass of Four or More Coronary Arteries	1.2	(0.9, 1.5)	1.2	(0.8,1.6)	1.6^**^	(1.1, 2.2)	0.6	(0.1, 2.2)
Coronary Bypass of Three Coronary Arteries	0.9	(0.8, 1.1)	0.8	(0.7,1.1)	1.1	(0.8, 1.4)	1.5	(0.7, 2.8)
Coronary Bypass of One Coronary Artery	1.0	(0.8, 1.1)	0.9	(0.7,1.1)	0.9	(0.7, 1.2)	1.6	(0.8, 3.0)
Open Replacement of Aortic Valve with Tissue Graft	1.5^**^	(1.1, 1.9)	1.7^***^	(1.2,2.2)	1.2	(0.8, 1.7)	1.5	(0.7, 3.1)
Open Replacement of Aortic Valve	1.0	(0.7, 1.3)	1.1	(0.8,1.6)	1.4	(0.9, 2.0)	0.6	(0.2, 1.9)
Diabetes mellitus without complication	0.8^**^	(0.7, 0.9)	0.9	(0.7,1.0)	0.8	(0.7, 1.0)	0.9	(0.5, 1.4)
Tobacco Use Disorder	0.8^**^	(0.7, 0.9)	0.7^**^	(0.6, 0.9)	0.6^***^	(0.5, 0.8)	0.9	(0.5, 1.6)
Atrial Fibrillation	1.0	(0.9,1.1)	1.0	(0.8,1.2)	1.0	(0.8, 1.2)	1.2	(0.7, 1.8)
Unspecified Hypertension	1.0	(0.9, 1.1)	1.1	(0.9,1.3)	1.1	(0.9, 1.3)	0.8	(0.5, 1.3)
Coronary Atherosclerosis	0.9	(0.7, 1.1)	0.7^*^	(0.5,0.9)	0.5^***^	(0.4, 0.7)	0.5	(0.3, 1.1)
Intermediate Coronary Syndrome	0.9	(0.8,1.0)	0.8	(0.7,1.0)	0.7	(0.5, 0.8)	0.7	(0.3, 1.2)
Hyperlipidemia	0.9	(0.8, 1.0)	0.8^*^	(0.7,0.9)	0.9	(0.7, 1.0)	0.6^*^	(0.4, 0.9)
Posthemorrhagic Anemia	0.7^***^	(0.6, 0.8)	0.8^*^	(0.7, 1.0)	0.9	(0.8, 1.1)	1.2	(0.8, 1.9)
Acute Myocardial Infarction	1.0	(0.9, 1.2)	0.8	(0.7,1.0)	0.7^**^	(0.6, 0.9)	0.7	(0.4, 1.1)
Congestive Heart Failure	1.0	(0.9,1.2)	1.2	(0.9,1.4)	1.1	(0.9, 1.3)	1.0	(0.6, 1.6)
Anemia, Unspecified	1.1	(0.9,1.2)	1.1	(0.9,1.4)	1.2	(0.9, 1.5)	0.9	(0.5, 1.6)
Pulmonary Collapse	0.9	(0.8, 1.1)	0.9	(0.7,1.1)	1.2	(0.9, 1.5)	1.2	(0.7, 1.9)
Acute Kidney Failure, Unspecified	0.9	(0.7, 1.1)	1.1	(0.8,1.3)	1.1	(0.8, 1.3)	1.9^**^	(1.2, 3.0)

*P* value notation: ^*****^: *p* ≤ 0.001; ^****^: 0.001 < *p* ≤ 0.01; ^***^: 0.01 < *p* ≤ 0.05; *no asterisk*: *p* > 0.05

## Abbreviations

AUC: Area under the Curve
CABG: Coronary artery bypass graft
CI: Confidence interval
CV: Cross validation
CVD: Cardiovascular disease
EHR: Electronic health record
FIM: Functional independence measure
HHC: Home health care
HIPAA: Health Insurance Portability and Accountability Act
ICD-9-CM: International Classification of Diseases, 9th Clinical Modification
ICU: Intensive care unit
IRB: Institutional Review Board
IRF: Inpatient rehabilitation facilities
LMA: Left against medical advice
LTCH: Long term care facilities
OSU-CHSI: Oklahoma State University Center for Health System Innovation
PAC: Post-acute care
ROC: Receiver operating characteristic
RR: Relative risk ratio
SNF: Skilled nursing facilities
VR: Valve replacement
